# Synthesis and Standardization of Outcomes in Severe Malaria Treatment Trials: Protocol for the Development of a Core Outcome Set (the COSSMaT Study)

**DOI:** 10.2196/78616

**Published:** 2026-04-13

**Authors:** Gideon Darko Asamoah, Sharon B Love, Diana M Gibb, Kathryn Maitland, Elizabeth C George

**Affiliations:** 1 Institute of Clinical Trials and Methodology, MRC Clinical Trials Unit at UCL, 90 High Holborn, London, WC1V 6LJ, United Kingdom, 44 07552098103; 2 Institute of Global Health Innovation, Department of Surgery and Cancer, Faculty of Medicine, Imperial College London, London, United Kingdom; 3 KEMRI Wellcome Trust Programme, Kilifi, Kenya

**Keywords:** severe malaria, core outcome sets, Delphi survey, clinical trials, treatment

## Abstract

**Background:**

Severe malaria remains a major global health challenge, particularly in endemic regions, where it causes substantial morbidity and mortality, especially among children and pregnant women. Although numerous clinical trials have evaluated treatments for severe malaria, heterogeneity in outcome selection, definition, and measurement limits comparability across studies, hampers evidence synthesis, and contributes to research waste. There is currently no established core outcome set (COS) for trials specifically focused on severe malaria treatment. Developing a COS is therefore essential to improve outcome standardization, strengthen evidence synthesis, and support evidence-based clinical practice.

**Objective:**

This study aims to develop a COS for trials evaluating the treatment of severe malaria.

**Methods:**

This protocol follows guidance from the Core Outcome Measures in Effectiveness Trials (COMET) Initiative and the Core Outcome Set–Standardised Protocol Items (COS-STAP) checklist. An updated systematic review of outcomes reported in randomized controlled trials of severe malaria treatment has been conducted. Searches covered the Cochrane Central Register of Controlled Trials (CENTRAL), MEDLINE via Ovid, and Literatura Latino-Americana em Ciências da Saúde (LILACS), as well as clinical trial registries, including the International Standard Randomised Controlled Trial Number (ISRCTN) registry, ClinicalTrials.gov, and the Pan-African Clinical Trial Registry (PACTR). Study selection followed PRISMA (Preferred Reporting Items for Systematic Reviews and Meta-Analyses) guidance. Outcomes important to patients and families are being identified through qualitative interviews with parents and caregivers of patients treated for severe malaria, with data analyzed using thematic synthesis. Outcomes identified across these sources will be prioritized through a 2-round, multistakeholder Delphi survey, with rounds separated by 4 weeks and a target sample of 60 to 80 participants. An online consensus meeting will then be held to agree on the final COS. Subsequently, outcome measurement instruments used in severe malaria trials will be identified and assessed, and a dissemination and implementation strategy will be developed to support uptake of the final COS.

**Results:**

Preliminary results from the updated systematic review identified 326 records through database and registry searches. Following screening and full-text review, 5 eligible randomized controlled trials conducted between 2020 and 2024 in Africa and Asia were included. Qualitative interviews with parents and caregivers have been conducted with 26 participants recruited and interviewed. The Delphi survey, consensus meeting, and outcome measurement instrument selection are ongoing and expected to be completed by September 2026.

**Conclusions:**

This protocol outlines a rigorous and inclusive approach to developing the first COS for severe malaria treatment trials. The resulting COS aims to improve outcome standardization, reduce reporting bias, and enhance the comparability and impact of future severe malaria trials.

## Introduction

### Background

Malaria persists as a formidable global health challenge, exacting a heavy toll on both morbidity and mortality rates worldwide [1-3]. Africa, in particular, bears a disproportionate burden, with 94% of malaria cases (246 million) and 95% (n=569,000) of malaria deaths recorded by the World Health Organization (WHO) in 2023 [2]. Children younger than 5 years constituted approximately 76% of malaria-related fatalities, making malaria a leading cause of childhood illness and death in the region [2,4]. Beyond its impact on children, malaria significantly influences maternal outcomes directly and neonatal outcomes indirectly because it heightens the risks of abortion, low birth weight, preterm delivery, stillbirth, and neonatal death [5-7].

The pursuit of improved malaria treatment remains an ongoing endeavor, particularly for severe disease [8,9]. However, a significant impediment arises from the lack of consensus on appropriate outcomes for use in clinical trials, thereby posing a challenge to measuring and evaluating the efficacy of antimalarial and/or adjunctive treatments. A systematic review has highlighted the considerable heterogeneity, with 101 diverse outcome measures reported in 27 trials spanning from 2010 to 2020 [10]. This disparity has led to the measurement of diverse outcomes and the use of various instruments for assessing the same outcome, resulting in inconsistencies in reported results and complicating the comparison of outcomes in systematic reviews and meta-analyses [10]. Creating a core outcome set (COS) for severe malaria treatment in trials could help mitigate the current deficiencies in addressing reporting challenges.

A COS is defined by the Core Outcome Measures in Effectiveness Trials (COMET) as “an agreed standardized set of outcomes that should be measured and reported, as a minimum, in all clinical trials in specific areas of health or health care” [11]. While a previous study has developed COS for malaria in the context of vaccine trials [12], a notable gap exists concerning the treatment of severe malaria, especially with the inclusion of adjunctive therapies [10]. This void is a critical concern for global health, given the severe and widespread impact of severe malaria. Urgent attention is warranted to address this gap.

The scope of the COS specifies the particular health care domain or condition it addresses, detailing its target population, interventions, and settings. This clarity ensures its appropriate and consistent use, thereby avoiding any ambiguity [13-15]. For this COS, the scope encompasses severe malaria, characterized by complications such as impaired consciousness, acidosis, and severe anemia. The target population includes all groups affected by severe malaria, including children under five, adults, pregnant women, and travelers who reside in endemic and nonendemic regions. The interventions involve antimalarial treatments and adjunctive therapies to manage complications. The setting is within clinical trials to standardize outcome selection and reporting, ensuring relevance and consistency across studies. The aim of the study is to develop a COS for trials in the treatment of severe malaria.

### Study Summary

We conducted a search for an existing COS for severe malaria treatment trials, adhering to the COMET guidelines’ recommendation to prevent duplication of ongoing or preexisting studies. No such study was found.

We will develop the COS through the following phases:

- Phase 1: develop a “long list” of outcomes to guide the creation of a COS for severe malaria treatment trials, we will use the following information sources:
  - Systematic review update: identify additional outcome sets reported for trials in the treatment of severe malaria
  - Consultation with clinical experts in severe malaria to incorporate recent and unpublished research insights
  - Qualitative study: identify severe malaria outcomes reported by patients, parents, and guardians or caregivers for measurement in treatment trials

- Phase 2: develop a preliminary COS (informed by the “long list” of outcomes from phase 1) with key stakeholders through a multistage (2- or 3-round, web-based) Delphi survey
- Phase 3: hold a consensus meeting with stakeholders to discuss and agree on the final COS for severe malaria treatment
- Phase 4: identify outcome measurement instruments for the outcome sets reported in severe malaria treatment trials
- Phase 5: develop a dissemination and implementation strategy for the final COS

## Methods

### Overview

**Figure 1.**
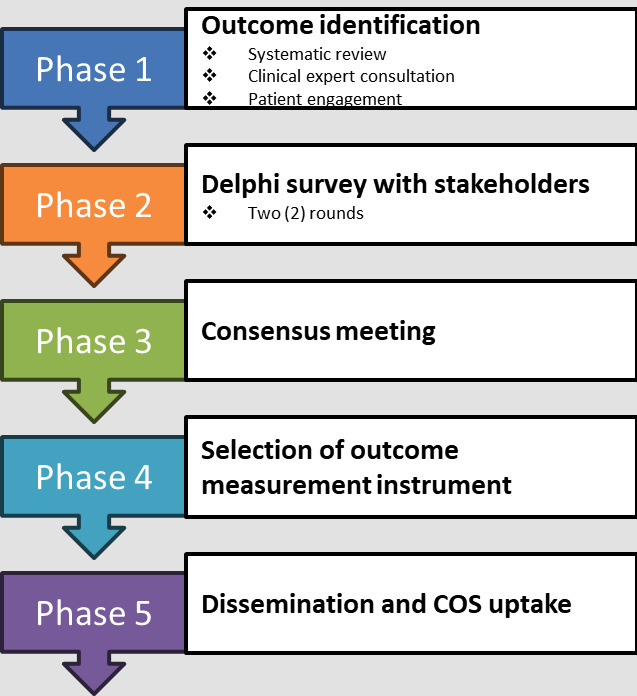
Schematic figure showing the overall workflow and link between the different phases. COS: core outcome set.

This study protocol will adhere to the guidelines outlined in the Core Outcome Set–Standardised Protocol Items (COS-STAP) statement checklist [16] (Checklist 1). The study has been registered on the COMET Initiative website [17]. The study will follow the COMET Initiative guidelines for developing a COS to produce a reliable, valid, and responsive minimum set of outcomes to be measured and reported in all clinical trials considering the treatment of severe malaria [13]. The schematic overall flow and link between the different phases of the study are presented in Figure 1.

### Phase 1: “Long List” of Outcomes to Guide the Creation of a COS for Severe Malaria Treatment Trials: Systematic Review Update

The systematic review of the study sought to build upon an earlier systematic review that examined outcomes reported in trials for severe malaria treatment conducted between January 1, 2010, and June 30, 2020 [10]. The objective of the update was to capture and integrate any newly published or registered trials focusing on severe malaria treatment conducted from July 1, 2020, to July 26, 2024. The updated systematic review followed the guidelines outlined in the “Reporting Systematic Reviews and Meta-Analyses of Studies That Evaluate Health Care Interventions” checklist (Checklist 2) [18].

### Eligibility Criteria

The eligibility criteria for this systematic review were established to ensure the inclusion of studies directly addressing the research objectives.

#### Inclusion Criteria

- Study design: Only randomized controlled trials (RCTs) conducted between 2020 and 2024 were included, representing the COS’s scope of use.
- Population: The review included studies focusing exclusively on hospitalized adults or children diagnosed with severe malaria, including cerebral malaria, based on the WHO criteria [19]. This population was chosen to provide insights into treatment efficacy and outcomes within the context of severe disease.
- Intervention and comparison: Eligible studies assessed the use of antimalarial therapies and adjunctive treatments for severe malaria. This criterion enabled a focused synthesis of evidence on therapeutic strategies for managing the condition.

#### Exclusion Criteria

- Studies involving animal models or nonhuman subjects
- Observational studies, literature reviews, and secondary analyses of RCTs
- Nonrandomized trials and research focusing on malaria prevention or vaccination
- Trials that included both severe and uncomplicated malaria without distinguishing between the two
- Conference abstracts, posters, or other non–peer-reviewed publications

### Search Strategy

The search strategy for this study was carefully designed to capture all relevant studies about severe malaria treatment within the defined scope. The search terms focused on 4 critical concepts: severe malaria, treatment, hospital, and RCTs. The search was restricted to English-language publications to ensure clarity and accessibility of the content. The time frame was deliberately chosen to provide an updated and comprehensive overview of outcomes from RCTs published between July 2020 and the search date. This time frame bridges the gap left by a prior systematic review [10], whose last search concluded in June 2020. The final search was conducted on July 26, 2024, covering publications up to that date. A complete list of the search terms and the detailed construction of the search strategy for MEDLINE, Literatura Latino-Americana em Ciências da Saúde (LILACS), and ClinicalTrials.gov are provided in Multimedia Appendix 1.

### Information Sources

Searches were conducted across key databases and clinical trial registries, including the Cochrane Central Register of Controlled Trials (CENTRAL), MEDLINE via Ovid, and LILACS, to ensure comprehensive geographical coverage. The search also extended to registries such as the International Standard Randomized Controlled Trial Number (ISRCTN), ClinicalTrials.gov, and the Pan-African Clinical Trial Registry (PACTR) to identify ongoing and unpublished trials, addressing potential publication bias. To enhance robustness, citation lists of retrieved trials were reviewed for additional records, ensuring no relevant studies were overlooked [20,21].

### Study Selection Process

**Figure 2.**
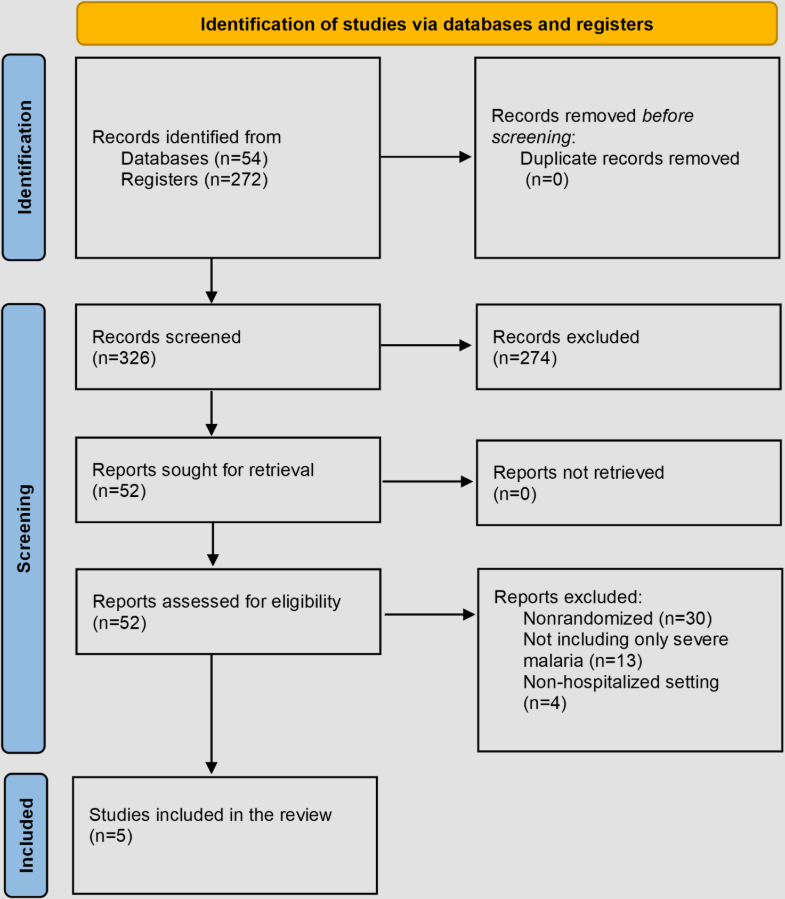
PRISMA (Preferred Reporting Items for Systematic Reviews and Meta-Analyses) diagram of included studies.

The search results were organized using EndNote X9 (Clarivate) reference management software. Duplicate entries were removed, followed by a double-screening of records. Titles and abstracts were initially reviewed to identify relevant trials, with eligible trials undergoing a detailed full-text review to ensure they met the inclusion criteria. For trials from registries without published results, entries were cross-referenced using key identifiers. We discussed and resolved any eligibility concerns by consensus. Trials not meeting the criteria were excluded, with explanations documented. The screening and selection process is detailed in a PRISMA (Preferred Reporting Items for Systematic Reviews and Meta-Analyses) flow diagram (Figure 2).

### Data Charting and Extraction

To systematically organize and analyze information, a data charting form was developed in Microsoft Excel (Multimedia Appendix 2). This form outlined specific variables to be collected, aligning with the study’s aim. The variables captured essential trial details, ensuring thorough data collection. The extracted data included trial characteristics such as study title, publication year, participant demographics, study design and phase, intervention type, and outcome measures. Outcome measures were described verbatim from study protocols or trial registry entries to maintain accuracy. Details about the instruments or methods used to measure these outcomes were also included, providing context for how the outcomes were quantified.

#### Consultation With Clinical Experts

Clinical experts were engaged to provide essential insights into practical and clinically relevant outcomes. Input was sought from clinical experts in the field of severe malaria, whose insights and familiarity with recent and unpublished research added an extra layer of comprehensiveness to the search strategy.

#### Qualitative Study

The COMET guidelines emphasize the importance of actively involving patients and the public throughout the development process to ensure that the selected outcomes are relevant and meaningful to those directly impacted [13]. By engaging with parents and caregivers, this qualitative research directly addresses the COMET Initiative guidelines on patient and public involvement [13]. Such engagement ensures that “all potentially relevant outcomes” are considered, thereby enhancing the comprehensiveness and relevance of the outcome list [22,23].

The study was conducted following the COREQ (Consolidated Criteria for Reporting Qualitative Research) guidelines (Checklist 3) [24] and the reporting recommendations for qualitative research methods in COS development outlined by Jones et al [25], as used in previous COS studies [22].

### Aim

The study aims to use qualitative research methods to explore and identify the outcomes of severe malaria that parents, guardians, and carers consider important to measure in treatment trials.

### Design

A descriptive qualitative research design was used in this study to identify outcomes reported by parents, guardians, and caregivers of children affected by severe malaria. Descriptive qualitative research aims to provide a detailed and comprehensive account of a phenomenon by focusing on the “who, what, where, and when” aspects, without delving into theoretical interpretations or complex explanations [26-28].

Without patient involvement in the COS development process, there is a risk that important outcomes will be overlooked, which could hinder the ability to assess treatment effectiveness. Several studies have used qualitative research in COS development to identify relevant outcomes, capture stakeholders’ perspectives, and ensure that the outcomes included in clinical trials reflect the priorities of patients, caregivers, and health care professionals [22,29-31]. Incorporating qualitative methods into the outcome identification process offers several benefits, particularly in providing a deeper understanding of why certain outcomes are important to patients, parents, and caregivers [22,32]. This helps ensure that the outcomes presented in the COS reflect the true priorities and needs of those most affected by severe malaria.

### Setting

Participants for this study were recruited from Ghana, a country in sub-Saharan Africa, which is one of the regions that bears a disproportionately high burden of malaria [33-35]. Targeting recruitment in these settings allowed for the inclusion of populations facing significant challenges with severe malaria, enriching our understanding of its impact. This study was conducted in Kumasi, the capital of Ghana’s Ashanti Region, a malaria hotspot with an increase in cases in recent years [36,37], with clinical malaria prevalence rates ranging from 3.4% to 16.9% [38]. The study was conducted at the Komfo Anokye Teaching Hospital (KATH) in Kumasi, within its Child Health Directorate. KATH was chosen for its role as the principal referral center for the region, providing specialized care for severe and complex conditions, including severe malaria, and offering access to a diverse patient population. The Child Health Directorate’s expertise in pediatric care enabled engagement with a broad range of stakeholders and grounded the research in a context reflecting the challenges and priorities of populations at the epicenter of the malaria burden.

### Recruitment Strategy

Participants were recruited from the Severe Malaria Africa: A Research and Trials Consortium (SMAART) observational study, which aims to describe the treatment for children with severe malaria from hospital presentation to 6 months after discharge. The study enrolled 2 cohorts: hospitalized children with severe malaria and time-matched controls with nonsevere malaria, with follow-up over 6 months to assess outcomes. Children aged 3 months to 15 years were included to generate insights into severe malaria management.

In total, 3 supervisors involved in this study are members of the SMAART Consortium. They facilitated contact with the local principal investigator at KATH, enabling the identification of key gatekeepers. These gatekeepers reviewed patient records to identify eligible participants, whose parents, guardians, or caregivers were then informed about the study and invited to participate.

### Sampling and Sample Characteristics

We initially planned to recruit parents, guardians, or caregivers of children currently receiving treatment for severe malaria at KATH. However, we revised our protocol to include those whose children had been treated for severe malaria within the past year. This change was made after observing that the severity of the children’s conditions often left their caregivers too distracted to engage fully in interviews. The 1-year time frame aimed to reduce recall bias [39,40]. Participants were purposefully sampled based on the following criteria: (1) aged ≥18 years; (2) parent and guardian or caregiver caring for an infant, diagnosed with and treated for severe malaria within a year at the time of recruitment; and (3) those admitted to and treated at the KATH during the time of recruitment.

### Data Collection Method

Experienced qualitative researchers, GDA and 2 SMAART study team members, conducted face-to-face semistructured interviews with parents, guardians, or caregivers. Semistructured interviews were chosen for their flexibility and ability to explore experiences, attitudes, and perceptions in depth [41,42]. This method allows researchers to guide discussions while adapting to participant responses, making it effective for collecting open-ended data and delving into personal and sensitive issues. The interviews were guided by a topic guide based on predefined themes from previous research, ensuring a thorough exploration of participant responses (Multimedia Appendix 3) [22,43,44].

Interviews were conducted in the local language to ensure participants felt comfortable. Consent was obtained before recording, respecting participants’ autonomy and privacy. The interviews took place in a private office within the Child Health Directorate, providing a confidential environment. During the interviews, demographic information of participants was collected, and a “concept-elicitation” approach encouraged open dialogue. Topics included understanding of severe malaria, important outcomes, hospital-based treatment experiences, and the broader impact of severe malaria on their lives. This approach ensured participants’ voices were central to the study’s findings.

### Management of Risk

The research team provided detailed explanations of the study procedures to potential participants, ensuring they had sufficient time to review the participant information sheet (PIS) and informed consent form (ICF) thoroughly. Participants were encouraged to ask questions and seek clarifications to ensure they fully understood the study’s purpose, requirements, and role. The research staff, all trained in ethical research practices, carefully assessed participants’ comprehension of the study procedures. Participants were informed that their participation was voluntary, with the right to withdraw at any time without affecting their medical care. A distress protocol adopted from a previous study (Multimedia Appendix 4) [22] was in place to support participants during potentially distressing interviews. If participants appeared uncomfortable, interviews were paused or stopped, and support options, such as referrals to health care professionals, were offered.

### Data Analysis

#### Overview

All interview recordings will be transcribed verbatim and translated into English. To ensure accuracy and preserve meaning, translations will be independently verified by a second researcher fluent in both languages. This verification process is essential for maintaining the integrity of participants’ accounts, particularly in relation to culturally specific meanings and idiomatic expressions [45-47].

NVivo software (version 14; Lumivero) [48] will be used to manage and organize the qualitative data. Each transcript will be treated as a distinct case, representing an individual participant [49]. Data analysis will be conducted using thematic synthesis. An inductive coding approach will be applied, comprising both semantic coding to identify outcomes explicitly reported by participants and interpretive coding to capture inferred concerns and underlying meanings [22,50].

To enhance rigor and consistency, 2 experienced qualitative researchers will independently code an initial subset of 10 transcripts. The coding framework will then be discussed and refined through iterative meetings before being applied to the remaining transcripts. This process will support analytical consistency and transparency across the dataset.

#### Generation of the “Long List” of Outcomes

In line with COMET guidance, all identified outcomes will be collated into a comprehensive long list and reviewed for duplication and conceptual overlap. Outcomes that are similar or closely related will be consolidated, and the refined list will be organized into broader outcome domains using the COMET-recommended outcome taxonomy for medical research [51], supported by frameworks applied in previous COS studies [10,52]. Domain allocation will be undertaken iteratively and refined through consultation with clinical experts to ensure clinical relevance, conceptual coherence, and consistency with the objectives of the COSSMaT (core outcome sets for severe malaria trials) study.

This structured and transparent approach to outcome identification and domain development will provide a robust foundation for subsequent Delphi surveys and consensus meetings, as recommended by COMET, and will support the development of a COS that is methodologically rigorous, stakeholder informed, and relevant to the evaluation of treatments for severe malaria.

### Phase 2: Delphi Survey With Stakeholders

#### Overview

The purpose of a Delphi survey is to collect opinions from a panel of participants, including experts in severe malaria and clinicians who regularly manage patients with severe malaria, on a specific topic to achieve group consensus [53]. Key stakeholders will be presented with plain language explanations of the “long list” of outcomes and will then rate the importance of each item for inclusion in the COS through a multistage Delphi survey.

#### Stakeholder Groups and Recruitment

Key stakeholders, including patient or support group representatives, health care workers, pharmaceutical industry representatives, policymakers, and researchers, will be involved in the COS development process. Stakeholders will be identified and recruited through expert networks associated with public health agencies, regulatory authorities, research institutions, pharmaceutical industries, and civil society organizations. Notable examples of such networks include the WHO, the Malaria Consortium, the SMAART Consortium, the COMET Initiative, Novartis, Cipla, support groups representing individuals with lived experiences, and first authors of related published articles.

We aim to promote the study and recruit stakeholders by developing a project webpage, featuring in network newsletters, presenting at seminars and conferences, using social media, and distributing leaflets. PIS and ICF will be provided electronically.

#### Multistage Delphi Survey

For the Delphi survey, a 2-round process will be conducted using the long list of outcomes derived from the identified information sources [13], with rounds separated by a 4-week interval. We will aim to recruit 60 to 80 participants overall, consistent with methodological evidence indicating that this sample size provides high replicability (≥80%) in multistakeholder Delphi surveys [54]. To ensure balanced representation, we will recruit at least 15 participants per stakeholder group. This distribution is intended to preserve meaningful comparison across groups and to maintain robust consensus thresholds in the event of attrition. Reminder emails and flexible completion windows will be used to support retention between rounds. Participants will have 4 weeks to complete each survey, with the option to save and resume their responses later. A third round may be considered depending on the analysis of the first 2 rounds. Descriptive statistics will be used to analyze overall scores for each stakeholder group, ultimately determining inclusion or exclusion of outcomes.

In round 1, participants will rate the importance of outcomes for inclusion in the COS using a 9-point scale, categorized as 9 to 7 (critically important), 6 to 4 (important but not critical), and 3 to 1 (not important). They can also briefly justify their choices, suggest changes, and propose additional items not covered in the survey. Suggested outcomes from the first round will be reviewed by the study committee for inclusion in the second round. Only responses from participants who rated at least 50% of items will be included.

In round 2, a summary of overall scores and scores from stakeholder groups on each outcome will be provided to participants along with their own scoring. Participants will be aware of others’ views but will not know individual scores to maintain anonymity, allowing unbiased expression of opinions. Participants can maintain or adjust their ratings in the second round if desired. Each round will have a 4-week completion time frame.

#### Consensus Definition

Outcomes that receive scores of 7 to 9 from 70% or more participants and scores of 1 to 3 from less than 15% of participants across all stakeholder groups will be considered for inclusion in the COS during the consensus meetings. Outcomes will be excluded if they are scored between 1 and 3 by at least 70% of participants across all stakeholder groups and between 7 and 9 by less than 15% of participants. Outcomes that do not fall into either category will be classified as having no consensus and will be discussed at the consensus meeting, as applied in other COS studies [55].

### Phase 3: Consensus Meetings

All participants who complete the Delphi process will be invited to participate in an online consensus meeting conducted via Zoom. To ensure that discussions reflect a range of perspectives while remaining manageable, each stakeholder group will be represented by a minimum of 2 participants. This approach is intended to balance inclusivity with feasibility and to ensure that all stakeholder voices are adequately represented during the outcome agreement. If an invited participant cannot attend, efforts will be made to find a suitable replacement from the same stakeholder group. To facilitate broad international participation, meetings will be scheduled to accommodate different time zones and participant availability.

Before the meetings, all necessary materials (outcomes rated through the Delphi process and voting patterns from different stakeholder groups) will be circulated to ensure participants are well prepared. To enhance engagement and decision-making, participants will be provided with a secure weblink to cast their votes remotely during the meeting.

The primary objective of the consensus meeting is to finalize the COS through structured discussions involving a diverse international panel. Outcomes identified through the Delphi process will be reviewed alongside voting patterns from different stakeholder groups. Discussions will be chaired by experienced, nonvoting facilitators to ensure neutrality. Following these discussions, participants will engage in anonymous, computerized voting. An outcome will be included in the final COS if it receives at least 70% approval from participants, with representation from all stakeholder groups.

### Phase 4: Outcome Measurement Instruments

Following agreement on the final COS, outcome measurement instruments for each outcome will be identified and evaluated in accordance with COSMIN guidelines[56]. This process will ensure that recommended instruments are valid, reliable, and appropriate for use in severe malaria treatment trials.

Systematic literature searches will be conducted to identify existing instruments used to measure the outcomes included in the COS. Sources will include published clinical trials and methodological studies relevant to severe malaria and related conditions. Identified instruments will then be assessed for methodological quality using the COSMIN Risk of Bias checklist, with the evaluation of key measurement properties such as validity, reliability, and responsiveness. Where evidence is insufficient or no appropriate instrument exists, gaps in outcome measurement will be highlighted, with recommendations for future research or instrument development.

### Phase 5: Dissemination Strategy

The results of this research will be written as part of a PhD dissertation and publications and will be disseminated at conferences, on the project webpage, featured in network newsletters, presented at seminars, as well as distribution of leaflets to all expert networks associated with the treatment of severe malaria in trials. To enhance accessibility, a plain English summary will be provided in infographics and on social media to facilitate broader engagement. The study protocol and results will be publicly available on the Open Science Framework to facilitate free and widespread sharing of the research. Participants will receive the findings of this research if they consent to it.

### Ethical Considerations

At the interview venue, participants were provided with a written ICF (Multimedia Appendix 5) after being guided through the PIS (Multimedia Appendix 6) to ensure they fully understood the study’s purpose and procedures. For participants unable to read or write, the PIS and ICFs were read aloud and translated into the local language to facilitate comprehension. Local gatekeepers played a crucial role in assisting participants by printing and signing the forms when necessary.

Participants unable to sign the consent form had the option to provide a thumbprint, ensuring inclusivity and respect for diverse literacy levels. All signed consent forms were securely stored in a locked filing cabinet at the local co-investigator’s office, safeguarding participants’ confidentiality and adhering to ethical research practices. All data were managed according to the University College London (UCL) Research Ethics Committee and the Komfo Anokye Teaching Hospital Institutional Review Board (KATH-IRB) guidelines.

Ethical approval for the qualitative interview phase of this study was obtained from the UCL Research Ethics Committee (project ID 27289/001) and the KATH-IRB, Ghana (reference KATH IRB/AP/087/24). All interview participants received written study information and provided written informed consent before participation. Data were treated with strict confidentiality, with participants assigned system-generated study identification numbers, and all personally identifiable information anonymized. No names or identifiable details appear in the thesis or resulting publications. Audio recordings and transcripts were securely stored on UCL OneDrive and accessible only to authorized members of the research team, with anonymized data retained for thesis write-up and repository deposit. Interview participants received GH¢50 (US $4.5) airtime to compensate for time, inconvenience, and transport costs.

The subsequent Delphi surveys and consensus meeting phases will be conducted under the same ethical approvals. Participants will be provided with written study information and will give informed consent before participation. Survey participants will be able to pause, save, and return to the survey, and participation will be voluntary with the option to withdraw at any time. Where applicable, names will be collected solely for acknowledgment and email addresses for survey administration and dissemination of study results; participants will be able to opt out of both. All data will be anonymized using study IDs, stored securely, and accessible only to authorized researchers. Email addresses will be retained until completion of the PhD project (anticipated September 30, 2026) and then deleted. No financial compensation is planned for participation in the survey or consensus meeting phases.

## Results

### Systematic Review Update

#### Study Selection

Following the update of the systematic review, a total of 326 studies were identified through database and trial registry searches aimed at capturing additional studies. After the initial screening of titles and abstracts, 272 studies were excluded for not meeting the inclusion criteria.

Of the 54 studies selected for full-text review, 5 were deemed eligible and met the predefined inclusion criteria. The inclusion process and exclusion rationale are comprehensively illustrated in Figure 2. This review builds upon a prior systematic review, which included 27 trials from an initial pool of 282 screened studies [10].

#### Study Characteristics

The included studies, conducted between 2021 and 2024, were geographically distributed across malaria-endemic regions in Africa (Mozambique, Malawi, Zambia, Nigeria, and Uganda) [57-60] and Asia (India) [61]. Participants were predominantly children, with ages ranging from 6 months to 17 years in 4 studies and 1 study in India focusing on adults aged 36 to 45 years. Sample sizes varied from 30 participants in a single-blind trial in India to 668 in a large double-blind trial in Malawi and Zambia [59,61].

All studies were RCTs with varying levels of blinding (single, double, and triple) [58-61] and research phases ranging from phases I or II [57,58] to phase III [60]. The interventions assessed were primarily adjunctive treatments to standard antimalarial therapies [57-59,61], with 1 study focusing exclusively on antimalarial treatment [60]. Primary outcomes varied, including mortality [60,61], pediatric cerebral malaria [59], acute kidney injury [57], and biomarkers such as angiopoietin-2 [58] (Table 1).

**Table 1.** Characteristics and results of sources of evidence.

Author	Year	Country	Participants	Sample size	Study design	Intervention type	Primary outcome
Varo et al [58]	2023	Mozambique	Children (aged 1‐12 y)	180	Phase IIb parallel arm, equally randomized, placebo-controlled, double-blind RCT^a^	Adjunctive treatment	Rate of decline in angiopoietin 2 from admission levels in children with SM^b^
Chilombe et al [59]	2022	Malawi, Zambia	Children (aged 2‐11 y)	668	Double-blinded, placebo-controlled, 2-armed RCT	Adjunctive treatment	Reduce T_max_ in pediatric CNS^c^ malaria and evaluate the “proof-of-concept” for potential neuroprotective efficacy
Alao et al [60]	2021	Nigeria	Children (aged 6 mo to 17 y)	235	Phase III triple-blinded, parallel RCT	Antimalarial	Number of deaths in 12, 24, and 48 h after randomization
Tiwari et al [61]	2023	India	Adults (aged 36-45 y)	30	Single-blind clinical trial	Adjunctive treatment	Death or discharge
Paasi et al [57]	2022	Uganda	Children (aged >6 mo to <12 y)	40	Phase I or II unblinded RCT	Adjunctive treatment	Correction of AKI^d^ at 48 h

a RCT: randomized controlled trial.

b SM: severe malaria.

c CNS: central nervous system.

d AKI: acute kidney injury.

### Qualitative Interview

The study involved 26 participants, the majority of whom were female (n=20, 76.9%), aged 21 to 70 years. The largest age group was 40 to 49 years (n=9, 34.6%), followed by 30 to 39 years (n=8, 30.8%).

Educational backgrounds varied: 50% (n=13) completed junior high school, and 7.7% (n=2) had no formal education.

Most participants were married (n=15, 57.7%) and mothers (n=16, 61.5%). Occupations included trading (n=16, 61.5%), skilled work (n=5, 19.2%), farming (n=3, 11.5%), professionals, and retired (n=1, 3.8% each). The patients were mostly male (n=16, 61.5%), aged 2 to 14 years, and older than 5 years (n=17, 65.4%), with the majority in primary school (n=14, 53.8%).

## Discussion

This study is expected to address a critical gap in malaria research by developing the first COS specifically for trials evaluating treatments for severe malaria. At present, there is no established COS for severe malaria treatment trials, and outcomes reported across studies vary widely in definition, measurement, and prioritization. The anticipated outcome of this work is a stakeholder-informed COS that provides a structured framework for the consistent selection and reporting of outcomes in future trials.

By standardizing outcomes and promoting the use of clearly defined and consistently measured end points, the proposed COS has the potential to improve comparability across severe malaria trials and strengthen the quality and transparency of evidence generation in this field. This standardization is expected to reduce outcome reporting bias and research waste while facilitating meaningful synthesis of findings across studies, including meta-analyses [62-64]. In the longer term, a COS for severe malaria treatment may support the selection of a single valid and reliable measurement instrument for each core outcome, thereby enhancing the interpretability and clinical usefulness of trial results within the broader malaria research landscape.

The methodological approach underpinning this protocol reflects current best practice in COS development by the COMET guidelines and in previous studies [11,16,65,66].

By integrating evidence from systematic reviews, qualitative research with parents and caregivers, and consensus methods involving multiple stakeholder groups, the study prioritizes inclusivity and relevance [10,63,67-70]. In particular, the explicit incorporation of parents and caregiver perspectives is expected to ensure that the resulting COS captures outcomes that reflect lived experience, functional impact, and quality of life, alongside traditional clinical endpoints [23,30,71,72]. This approach aligns with contemporary efforts to make clinical research more patient centered and responsive to stakeholder priorities.

Several limitations are acknowledged at the protocol stage. The qualitative component is being conducted within a single malaria-endemic country, which may limit the diversity of perspectives captured and may not fully reflect the experiences and priorities of patients and caregivers in other endemic regions. Furthermore, due to resource constraints, the Delphi survey and consensus processes will be conducted in English only, which may restrict participation from some stakeholders and reduce inclusivity across non–English-speaking settings. The findings will depend on the successful recruitment and sustained engagement of diverse stakeholder groups throughout the Delphi and consensus stages. As with all consensus-based methodologies, the final COS will represent areas of agreement among participants and may not capture all context-specific priorities relevant to every setting.

Future work following completion of the COS will focus on dissemination and implementation. Planned dissemination includes publication in peer-reviewed journals, presentation at relevant conferences, and sharing findings with study participants and the wider malaria research community. Uptake of COS in future trials will be critical to realizing its intended benefits, and engagement with trialists, funders, and guideline developers will be important to support adoption.

## Supplementary material

10.2196/78616 Multimedia Appendix 1 Search strategy for MEDLINE and LILACS.

10.2196/78616 Multimedia Appendix 2 Data chart.

10.2196/78616 Multimedia Appendix 3 Interview (topic) guide.

10.2196/78616 Multimedia Appendix 4 Distress protocol for interviews.

10.2196/78616 Multimedia Appendix 5 Informed consent form.

10.2196/78616 Multimedia Appendix 6 Participant information leaflet.

10.2196/78616 Checklist 1 PRISMA checklist.

10.2196/78616 Checklist 2 The COS-STAP statement checklist.

10.2196/78616 Checklist 3 COREQ checklist.

## Abbreviations

CENTRAL: Cochrane Central Register of Controlled Trials
COMET: Core Outcome Measures in Effectiveness Trials
COREQ: Consolidated Criteria for Reporting Qualitative Research
COS: core outcome set
COS-STAP: Core Outcome SetStandardized Protocol Items
COSSMaT: core outcome sets for severe malaria trials
ICF: informed consent form
ISRCTN: International Standard Randomized Controlled Trial Number
KATH-IRB: Komfo Anokye Teaching Hospital Institutional Review Board
LILACS: Literatura Latino-Americana em Ciências da Saúde
PACTR: Pan-African Clinical Trial Registry
PIS: participant information leaflet
PRISMA: Preferred Reporting Items for Systematic Reviews and Meta-Analyses
RCT: randomized controlled trial
SMAART: Severe Malaria Africa - A Consortium for Research and Trials
UCL: University College London
WHO: World Health Organization
