# Supplementary material for: Synthesis and Standardization of Outcomes in Severe Malaria Treatment Trials: Protocol for the Development of a Core Outcome Set (the COSSMaT Study)
Source: JMIR Res Protoc. 2026 Apr 13;15:e78616. doi: 10.2196/78616 (PMC13075636; doi:10.2196/78616)
Supplement: Multimedia Appendix 1 [file resprot-v15-e78616-s001.docx]

## Search strategy for Medline, and LILACS

Medline (Ovid) search

| **#** | **Searches** | **Results** |
| --- | --- | --- |
| 1 | Malaria/dt, th [Drug Therapy, Therapy] | 11736 |
| 2 | Malaria, Cerebral/dt, th [Drug Therapy, Therapy] | 553 |
| 3 | Malaria, Falciparum/dt, th [Drug Therapy, Therapy] | 7051 |
| 4 | Malaria, Vivax/dt, th [Drug Therapy, Therapy] | 1360 |
| 5 | (severe adj3 malaria).tw. | 5537 |
| 6 | 1 or 2 or 3 or 4 or 5 | 23437 |
| 7 | treatment*.mp. [mp=title, abstract, original title, name of substance word, subject heading word, floating sub-heading word, keyword heading word, organism supplementary concept word, protocol supplementary concept word, rare disease supplementary concept word, unique identifier, synonyms] | 6584392 |
| 8 | therap*.mp. [mp=title, abstract, original title, name of substance word, subject heading word, floating sub-heading word, keyword heading word, organism supplementary concept word, protocol supplementary concept word, rare disease supplementary concept word, unique identifier, synonyms] | 7682765 |
| 9 | "drug therap*".mp. [mp=title, abstract, original title, name of substance word, subject heading word, floating sub-heading word, keyword heading word, organism supplementary concept word, protocol supplementary concept word, rare disease supplementary concept word, unique identifier, synonyms] | 2838878 |
| 10 | "drug treatment*".mp. [mp=title, abstract, original title, name of substance word, subject heading word, floating sub-heading word, keyword heading word, organism supplementary concept word, protocol supplementary concept word, rare disease supplementary concept word, unique identifier, synonyms] | 56900 |
| 11 | 7 or 8 or 9 or 10 | 10726309 |
| 12 | 6 and 11 | 20842 |
| 13 | "randomized controlled trial".pt. | 624428 |
| 14 | (random$ or placebo$ or single blind$ or double blind$ or triple blind$).ti,ab. | 1675386 |
| 15 | (retraction of publication or retracted publication).pt. | 49435 |
| 16 | 13 or 14 or 15 | 1826051 |
| 17 | (animals not humans).sh. | 5238019 |
| 18 | ((comment or editorial or meta-analysis or practice-guideline or review or letter) not "randomized controlled trial").pt. | 5805531 |
| 19 | (random sampl$ or random digit$ or random effect$ or random survey or random regression).ti,ab. not "randomized controlled trial".pt. | 141798 |
| 20 | 16 not (17 or 18 or 19) | 1323785 |
| 21 | 12 and 20 | 1912 |
| 22 | limit 21 to (english language and yr="2020 -Current") | 260 |
| 23 | (Hospital* or inpatient*).tw. | 1823913 |
| 24 | limit 23 to (english language and yr="2010 -Current") | 540842 |
| 25 | 21 and 23 and 24 | 37 |
|  |  |  |

LILACS Database search strategy

Search term: malaria (title, abstract, subject)

Type of study: Controlled clinical trial

Language: English

Search Results: 17

Publication year range: 2020-2024

ClinicalTrials.gov search strategy

(Severe OR cerebral OR falciparum OR vivax)

AND

(hospital OR patient OR inpatient)

AND

(treatment OR therapy OR drug OR medication)

NOT

(uncomplicated)

NOT

(prevention OR vaccine OR diagnostic test OR net)

Category: Interventional studies

Search Results: 272

Start date 01/07/2020 to 26/07/2024
