# Supplementary material for: Synthesis and Standardization of Outcomes in Severe Malaria Treatment Trials: Protocol for the Development of a Core Outcome Set (the COSSMaT Study)
Source: JMIR Res Protoc. 2026 Apr 13;15:e78616. doi: 10.2196/78616 (PMC13075636; doi:10.2196/78616)
Supplement: Multimedia Appendix 2 [file resprot-v15-e78616-s002.docx]

## Data Chart

| Author | Year | Country | Participants/ Sample size | Study design | Intervention  type | Primary outcome | Secondary outcome |
| --- | --- | --- | --- | --- | --- | --- | --- |
|  |  |  |  |  |  |  |  |
|  |  |  |  |  |  |  |  |
|  |  |  |  |  |  |  |  |
|  |  |  |  |  |  |  |  |
|  |  |  |  |  |  |  |  |
