# Supplementary material for: Synthesis and Standardization of Outcomes in Severe Malaria Treatment Trials: Protocol for the Development of a Core Outcome Set (the COSSMaT Study)
Source: JMIR Res Protoc. 2026 Apr 13;15:e78616. doi: 10.2196/78616 (PMC13075636; doi:10.2196/78616)
Supplement: Multimedia Appendix 3 [file resprot-v15-e78616-s003.docx]

**Interview (Topic) Guide**

**Title of Study:** Core Outcome Set for trials in the treatment of severe malaria. A qualitative study of patients, parents, and guardians/caregivers-reported outcomes for severe malaria treatment.

**Department:** MRC Clinical Trials Unit at UCL, Institute of Clinical Trials and Methodology

**Name and Contact Details of the Researcher(s):** Mr Gideon Darko Asamoah, Professor Diana Gibb, Dr Elizabeth George, Dr Sharon Love, Dr Marthe Le Prevost, all of University College London (UCL), UK, Professor Daniel Ansong of Kwame Nkrumah University of Science and Technology, Ghana, and Professor Kathryn Maitland of Imperial College London and KEMRI-Wellcome Trust Research Programme, Kenya.

**Name and Contact Details of the Principal Researcher:** Gideon Darko Asamoah, gideon.asamoah.23@ucl.ac.uk

**Name and Contact Details of the UCL Data Protection Officer:** data-protection@ucl.ac.uk

**This study has been approved by the UCL Research Ethics Committee: Project ID number:**

Interview Outline

I. Introduction

II. The participant will receive a reminder regarding the study's purpose.

III. The participant will be allowed to ask questions about the study.

IV. The participant will be asked if they are still willing to take part.

V. The participant will be asked if they consent to audio/video recording of the interview.

VI. They will be requested to complete the consent form, sign it, and hand it over to the interviewer.

VII. In the case of teleconference interviews, participants will be reminded to send the signed consent form via email before the video/audio interview begins.

VIII. The specific questions will be asked of the participant.

*I. Introduction*

Thanks for joining our interview today! We're grateful for your participation in our study, which is part of a PhD project developing a "Core Outcome Set" for severe malaria trials. Feel free to ask for more details. Your participation is entirely voluntary, and you can withdraw without giving a reason. Take your time deciding if you want to be part of the study. Thanks for your interest and for reading through this information sheet.

*II. The participant will receive a reminder regarding the study's purpose.*

In this research we will use qualitative research to explore and better understand peoples’ /or their children’s experiences of having severe malaria. This will better help us understand what it is like to have severe malaria and identify crucial outcomes for assessing severe malaria treatment in future trials. The research involves incorporating reported outcomes from patients, parents, and guardians/caregivers regarding severe malaria. This is part of PhD research project that aims to examine the standardisation of outcomes reported in severe malaria trials through the development of a Core Outcome Set based on informed input.

*III. The participant will be allowed to ask questions about the study.*

Before we get started, do you have any questions?

*IV. The participant will be asked if they are still willing to take part.*

Before we proceed, we want to ensure that you are still comfortable participating in this interview. If you feel the need for a break or find any questions challenging to answer, please feel free to let us know at any point.

V. The participant will be asked if they consent to audio recording of the interview.

VI. They will be requested to complete the consent form, sign it, and hand it over to the interviewer.

VII. In the case of teleconference interviews, participants will be reminded to send the signed consent form via email before the video/audio interview begins.

VIII. The specific questions will be asked of the participant.

| 1 | *What do you believe is the reason you/your child/relative was being admitted to the hospital?* |
| --- | --- |
| 2 | *Are you aware of the diagnosis given to you/your child/relative? Could you provide some details or explanation about it?* |
| 3 | *Which treatment options were offered you/your child/relative and how did you decide which treatment to have/give to your child?*  What specific information did the patient, parent/guardian, or caregiver seek regarding the treatment their child would receive, and what factors influenced their decision-making process when considering treatment options? |
| 4 | *What kind of treatment was administered to you/your child/relative during their hospital stay?* |
| 5 | *What kind of treatment did you/your child/relative receive after being discharged from the hospital?* |
| 6 | *What impact has the treatment had or is currently having?*  Prompt areas such as physical health, mental health, effects on family and relationships, developmental milestones, etc.  Ask if the patient, parent/guardian, or caregiver could share what might be considered the most challenging side effect their infant has encountered? |
| 7 | *Are you, your baby, or your relation experiencing any persistent illnesses or issues? If so, could you describe them?* |
| 8 | *What concerns do you have for yourself, your child, or your relation’s future?* |
| 9 | *Is there any information that you wish you had received during your time in the hospital but did not get?*  *If so, what specific information would you have liked to receive?* |
| 10 | *Did the explanations and information provided about the treatment you, your child, or your relation received match your actual experience?*  Ask if the patient, parent/guardian, or caregiver was provided adequate information regarding treatment offered to them and if they were satisfied with the information. |
| 11 | *Is there any information or advice from your healthcare provider that you disagree with?* |
| 12 | *Is there any information or advice from your healthcare provider that you consider crucial?* |
| 13 | *If I mention that I am studying health outcomes, what does the term "health outcome" mean to you?*  *How would you describe an outcome in the context of health?*  what they can expect in terms of improvement or recovery after seeing a doctor or getting treatment. |
| 14 | *In your opinion, what outcomes do you believe are crucial to measure to assess the health progress of you, your child, or your relation?* |
| 15 | *What is most important to you regarding the health of you, your child, or your relation?* |
| 16 | *How does your health, your child, or your relation impact you personally?* |
| 17 | *How does your health, your child, or your relation impact other members of your family?* |
| 18 | *Researchers and doctors have explored (insert list of outcomes derived from systematic review).*  *What are your thoughts on this?*  *Is there anything else you would like to contribute or mention?* |
