# Supplementary material for: Synthesis and Standardization of Outcomes in Severe Malaria Treatment Trials: Protocol for the Development of a Core Outcome Set (the COSSMaT Study)
Source: JMIR Res Protoc. 2026 Apr 13;15:e78616. doi: 10.2196/78616 (PMC13075636; doi:10.2196/78616)
Supplement: Multimedia Appendix 4 [file resprot-v15-e78616-s004.docx]

**Distress Protocol for Interviews (adopted from the COHESION study).**

| Signs that may suggest stress during the interview procedure | Procedure when participant displays signs of distress and questions to determine level of distress | Response or behaviour of participants in response to questions | Is the participant displaying signs of strong emotional distress and/or is there a concern for their safety?  (YES/NO) | Is there a cause to believe the participant may be in danger?  (YES/NO) |
| --- | --- | --- | --- | --- |
| Participant informs the interviewer that they are experiencing a high level of distress. | 1. Stop the interview.  2. Offer support to the participants and allow them time to recover.  3. Determine the level of distress through the following questions:  a. Would you like to share what you are thinking?  b. Would you like to share how you are feeling?  c. Do you feel you can continue with this interview?  d. Would you like to end this interview?  e. Do you feel you would be able to go on about your day as normal after this interview?  (For Interviewer): Do you think that the participant is experiencing emotional distress beyond what would be expected from this interview? |  |  |  |
| A participant shows signs of emotional distress (e.g. crying, becoming agitated, loss of concentration, etc.) | 1. Stop the interview.  2. Offer support to the participants and allow them time to recover.  3. Determine the level of distress through the following questions:  a. Would you like to share what you are thinking?  b. Would you like to share how you are feeling?  c. Do you feel you can continue with this interview?  d. Would you like to end this interview?  e. Do you feel you would be able to go on about your day as normal after this interview?  4. (For Interviewer): Do you think that the participant is experiencing emotional distress beyond what would be expected from this interview? |  |  |  |
| Actions for the Interviewer:  1. If the distress displayed by the participant is what would be expected in an interview on this sensitive  topic;  offer the participant support and the option to:  a. Stop the interview.  b. Give the participant a break from the interview.  c. Continue the interview.  2. If the distress displayed by the participant is beyond what would be expected in an interview such as this on a sensitive topic;  a. Encourage the participant to follow up with his/her GP or visit the local hospital for support.  b. Provide the participant with the number of the emergency room at the nearest hospital (location specific) and encourage the participant to contact this service if their distress is increased in the hours/days/weeks following the interview.  c. Indicate that you (the interviewer), with the participant’s permission, will follow up with/ contact the participant on the day following the interview.  d. Notify the PI/Lead Researcher of the steps undertaken and the recommendations given to the participant.  3. If the distress displayed by the participant indicates they may be in immediate danger;  a. Contact the local authorities, unless a family member can transport the participant to the nearest hospital.  b. Indicate that you (the interviewer), with the participant’s permission, will follow up with/ contact the participant on the day following the interview.  c. Notify the PI/Lead Researcher and the hospital clinical team of the steps undertaken and the recommendations given to the participant. | | | | |
