# Supplementary material for: Synthesis and Standardization of Outcomes in Severe Malaria Treatment Trials: Protocol for the Development of a Core Outcome Set (the COSSMaT Study)
Source: JMIR Res Protoc. 2026 Apr 13;15:e78616. doi: 10.2196/78616 (PMC13075636; doi:10.2196/78616)
Supplement: Multimedia Appendix 5 [file resprot-v15-e78616-s005.docx]

**Informed Consent Form**

**Statement of person obtaining informed consent:**

I have fully explained this research to ____________________________________ and have given sufficient information about the study, including that on procedures, risks and benefits, to enable the prospective participant to make an informed decision to or not to participate.

DATE: _____________________ NAME: _________________________________

**Statement of person giving consent:**

I have read the information on this study/research or have had it translated into a language I understand. I have also talked it over with the interviewer to my satisfaction.

I understand that my participation is voluntary (not compulsory).

I know enough about the purpose, methods, risks, and benefits of the research study to decide that I want to take part in it.

I understand that I may freely stop being part of this study at any time without having to explain myself and that this will have no impact on the clinical care I receive.

I have received a copy of this information leaflet and consent form to keep for myself.

NAME:_________________________________________________________________

DATE: ____________ SIGNATURE/THUMB PRINT: ___________________

**Statement of person witnessing consent (Process for Non-Literate Participants):**

I (Name of Witness) certify that information given to

(Name of Participant), in the local language, is a true reflection of what l have read from the study Participant Information Leaflet, attached.

WITNESS’ SIGNATURE (maintain if participant is non-literate): ____________________

MOTHER’S SIGNATURE (maintain if participant is under 18 years): ________________

MOTHER’S NAME: ______________________________________________________

FATHER’S SIGNATURE (maintain if participant is under 18 years): _________________

FATHER’S NAME: _____________________________________________________
