# Supplementary material for: Synthesis and Standardization of Outcomes in Severe Malaria Treatment Trials: Protocol for the Development of a Core Outcome Set (the COSSMaT Study)
Source: JMIR Res Protoc. 2026 Apr 13;15:e78616. doi: 10.2196/78616 (PMC13075636; doi:10.2196/78616)
Supplement: Multimedia Appendix 6 [file resprot-v15-e78616-s006.docx]

**Participant Information Leaflet**

**This leaflet must be given to all prospective participants to enable them to know enough about the research before deciding to or not to participate**

**Title of Research:** Core Outcome Set for trials in the treatment of severe malaria. A qualitative study of patients, parents, and guardians/caregivers-reported outcomes for severe malaria treatment.

**Name(s) and affiliation(s) of researcher(s):**

This study is being conducted by Mr Gideon Darko Asamoah, Professor Diana Gibb, Dr Elizabeth George, Dr Sharon Love, Dr Marthe Le Prevost, all of University College London (UCL), UK, Professor Daniel Ansong of Kwame Nkrumah University of Science and Technology, Ghana, and Professor Kathryn Maitland of Imperial College London and KEMRI-Wellcome Trust Research Programme, Kenya.

**Background (Please explain simply and briefly what the study is about):**

Malaria remains a significant global health challenge, particularly affecting Africa, where 608,000 deaths were reported in 2020, with children under five accounting for 78% of fatalities. While progress has been made in prevention and vaccine development, the lack of agreement on outcomes (results of treatment) in severe malaria trials makes it more difficult to evaluate available treatment. A review of 27 severe malaria trials carried out between 2010 and 2020 found that many different outcomes were measured (101 in total) which makes it very hard to compare findings from studies.

Addressing this gap through a COS would ensure the same outcomes are used across different trials. It will also make research clearer and more open, and support evidence-based treatment guidelines, ultimately reducing the global health burden of severe malaria.

**Purpose(s) of research:**

In this research we will use qualitative research to explore and better understand peoples’ /or their children’s experiences of having severe malaria. This will better help us understand what it is like to have severe malaria and identify crucial outcomes for assessing severe malaria treatment in future trials. The research involves incorporating reported outcomes from patients, parents, and guardians/caregivers regarding severe malaria.

This is part of PhD research project that aims to examine the standardisation of outcomes reported in severe malaria trials through the development of a Core Outcome Set based on informed input.

**Procedure of the research, what shall be required of each participant and approximate total number of participants that would be involved in the research:**

We are going to gather information about the results (outcome measures) of trials for severe malaria, both those already reported and those yet to be reported, by reviewing existing studies and outcomes reported by patients, their parents, and guardians/caregivers respectively.

To make sure that views from patients, parents, and guardians/caregivers regarding severe malaria are included in this research, we are using qualitative research methods, which allows us to explore the stories shared by patients and those caring for them and identify outcomes important to them. For this part of the research, we plan to recruit 30 participants. People can take part if they are aged 18 and above or are parents, or other family members responsible for caring for infants diagnosed with and treated for severe malaria. Caregivers should be individuals with relevant experience in caring for patients with severe malaria, and they must all be 18 years or older.

The information we collect will help us create a long list of outcomes. We will then ask key groups of people (stakeholders) to agree a shorter list of the most important outcomes that can be reported on in all severe malaria trials in the future. Key stakeholders will include clinical experts, patient/patient representatives, healthcare workers, pharmaceutical industry representatives, policymakers, and researchers. Firstly, key stakeholders will complete a series of surveys starting in 2024 and 2025. Following the surveys, a consensus meeting will be held with the key stakeholders during which the essential list of outcomes for severe malaria treatment trials will be finalised. Subsequently, we will hold another meeting with clinical experts and researchers to choose a single, reliable method for measuring each outcome in the list.

**Risk(s):**

During the interviews, sensitive subjects may arise that could be distressing. You are not obliged to discuss any uncomfortable topics. You can withdraw at any time. If you do withdraw from the study, this will not affect the care you are receiving in the hospital in any way. If feel you upset during the interview, we will pause or halt the interview and discuss options for seeking support, such as professionals or support services, will be discussed. We will give you a list of support services. Additionally, we have established a protocol to address distressing situations during interviews, particularly when recalling events that may cause distress.

**Benefit(s):**

We are working to standardise outcomes reported in severe malaria trials through this project. Establishing a Core Outcome Set (COS) for severe malaria treatment ensures precise and consistent outcome definitions. Involving patients and the public in developing COS provides valuable perspectives for identifying important outcomes, prioritising patient needs and experiences during the Delphi survey and consensus meeting. Standardisation is crucial for comparing studies, improving research quality.

**Confidentiality:**

Any information gathered about you during this research will be treated with the utmost confidentiality. You will be identified with a study ID, ensuring the anonymity of your data. No names will be included in the thesis or any subsequent publications, and all identifiable demographic or personal details will be omitted to safeguard anonymity. Only authorized members of the research team will have access to the database and any data collected from you, preventing any link between your answers and your identity. Please note that assurances on confidentiality will be strictly adhered to unless evidence of wrongdoing or potential harm is uncovered. In such cases, the University may be obliged to contact relevant statutory bodies/agencies.

**Voluntariness:**

Taking part in this study should be out of your own free will. You are not under obligation to take part in the research. Participation is entirely voluntary.

**Alternatives to participation:**

If you choose not to participate, this will not affect your treatment in this hospital/institution in any way.

**Withdrawal from the research:**

You retain the right to withdraw at any time, without providing a reason and without facing any adverse consequences. However, once you withdraw, any data already collected through the survey cannot be retracted. You may also choose not to answer any question you find uncomfortable or private.

**Consequence of Withdrawal:**

You retain the right to withdraw at any time, without providing a reason and without facing any adverse consequences. However, once you withdraw, any data already collected through the survey cannot be retracted. You may also choose not to answer any question you find uncomfortable or private.

**Costs/Compensation:**

For your time/inconvenience/transport to the hospital, we will compensate you with GH¢50 airtime (mobile provided of your choice) to show our appreciation for your participation).

**Contacts:**

Should you need further assistance about this study please see the contact details below:

PhD Student: Gideon Darko Asamoah [gideon.asamoah.23@ucl.ac.uk](mailto:gideon.asamoah.23@ucl.ac.uk)

PhD supervisor: Elizabeth George [elizabeth.george@ucl.ac.uk](mailto:elizabeth.george@ucl.ac.uk)
